# Orthogonal Experimental Study on the Factors Affecting the Mechanical Properties of Alkali-Activated Slag Materials

**DOI:** 10.3390/ma15248795

**Published:** 2022-12-09

**Authors:** Kai Zhang, Haifeng Lu, Jie Li, Hao Bai

**Affiliations:** 1 School of Civil Engineering, Wuhan University, Wuhan 430072, China; 2 Hubei Key Laboratory of Geotechnical and Structural Engineering Safety, Wuhan University, Wuhan 430072, China; 3 CISDI ENGINEERING CO., LTD., Chongqing 401122, China; 4 Brisight (Hainan) Technology and Development CO., LTD., Haikou 570216, China

**Keywords:** blast furnace slag, alkali-activated, uniaxial compressive strength, orthogonal experimental, elemental composition

## Abstract

Blast furnace slag is one of the largest solid wastes in the world. The slag-based geopolymer obtained by alkali activation has many advantages, such as a high strength, a good corrosion resistance, low carbon and environmental protection. Existing studies have shown that the mechanical properties of slag-based geopolymers are related to the combined effects of many factors, but there is still a lack of reliable conclusions on the primary and secondary influence degree of each factor, which greatly affects the scientific preparation and application of slag-based geopolymers. In order to solve this problem, we choose to proceed from the two perspectives of the mix ratio of the alkali activator and the elemental composition of raw materials. Through the orthogonal analysis method, this paper studies the influence of the modulus of the alkali activator, the solid-to-liquid ratio of the activator, the water–cement ratio and the metakaolin replacement rate on the uniaxial compressive strength of a slag-based geopolymer. The results show that when the solid–liquid ratio is about 0.25, the modulus of the alkali activator is 1.3~1.5, the water–cement ratio is about 0.4 and the samples with higher strength can be prepared. With the addition of metakaolin, a new gel phase NASH was formed in the system, which significantly promoted the late strength and toughness growth of the sample. The research results comprehensively analyze the influence of different factors on the mechanical properties of the slag-based geopolymer, which can provide a valuable reference for the engineering application of alkali-activated slag materials.

## 1. Introduction

Blast furnace slag is a solid waste produced in the process of blast furnace ironmaking. At present, the global annual output has exceeded 250 million tons [1]. In many developing countries, the comprehensive utilization rate of blast furnace slag is still at a low level, which seriously restricts the sustainable development of local economy and society [2,3]. The slag-based geopolymer obtained by alkali activation has many advantages such as a high strength, a good corrosion resistance, low carbon and environmental protection [4,5,6]. It has good application prospects in the development trend of green cementitious materials in the future and has become the mainstream research direction of slag resource utilization [7,8,9].

The sources of raw materials for geopolymers are very wide. According to the elemental composition, they can be divided into two categories: high-calcium and low-aluminum raw materials, represented by slag and coal gangue, and high-aluminum and low-calcium raw materials, represented by metakaolin and activated sediment [10,11]. Among them, the factors affecting the uniaxial compressive strength of alkali-activated high-calcium materials are very complex, and a large number of studies have reached inconsistent conclusions [12,13,14]. Representatively, Nath et al. found that when the high-calcium raw material slag was added to the fly ash-based polymer, the strength of the sample increased first and then decreased with the increase in the calcium content in the system [15]. Luo Xinchun et al. found that the addition of CaO can promote the formation of trigonal calcite in the reaction system, significantly improve the early strength of the geopolymer samples and shorten the setting time [16]. Cui Chao et al., through the experiment, think that the strong alkaline activator with a lower modulus can promote the geological polymerization reaction of low-calcium precursors, while the weak-alkaline activator with a higher modulus is beneficial to the hydration reaction of the alkali-activated high-calcium precursors [17]. In addition, the selection and mix ratio of the alkali activator also have a key influence on the strength of alkali slag samples [18,19]. By testing the uniaxial compressive strength of a red mud-slag-based geopolymer, Ding Zhu et al. found that the activation effect of water glass on the raw materials is better than that of sodium hydroxide. At the same time, the higher alkali content will have an adverse effect on the alkali activation reaction [20]. Peng Hui et al. found that water consumption had little effect on the mechanical strength of geopolymer materials but had a great influence on the setting time [21]. For alkali-activated slag cementitious materials, Chi et al. believed that the optimal modulus of the alkali activator was about 0.8, while the optimal modulus obtained by Chen et al. was around 1.5. There is a great difference between the two conclusions [22,23].

In summary, the existing studies have shown that the mechanical properties of slag-based geopolymers are related to the combined effects of many factors [24,25]. However, the mixture of many factors affected our judgment on the dominant factors of the geopolymer mechanical properties, resulting in many inconsistent conclusions. This has hindered the scientific preparation and popularization of slag-based geopolymers [26,27]. In this paper, we choose to proceed from the two perspectives of the mix ratio of the alkali activator and the elemental composition of raw materials. Through the orthogonal analysis method, this paper scientifically evaluates the influence of the modulus of the alkali activator, the solid-to-liquid ratio of the activator, the water–cement ratio and the metakaolin replacement rate on the uniaxial compressive strength of slag-based geopolymers. The research conclusion explains the influence mechanism of various factors on the mechanical strength of slag-based geopolymers, which can provide some reference for the scientific preparation and application of alkali-activated slag materials.

## 2. Sample Preparation and Characterization Methods

### 2.1. Materials

(a) Blast furnace slag (BFS) is a molten material with silicate and aluminosilicate as the main components after quenching and granulation when smelting pig iron in the blast furnace [10]. The BFS was provided by Dehang mineral product processing plant in Hebei Province, China. It is an S105 grade mineral powder. This gray powder has a specific surface area of 480 m^2^/kg, a density of 3.1 g/cm^3^, and a loss on ignition of 0.84%. The X-ray fluorescence spectrum showed that the BFS contains many active components, such as SiO_2_ and Al_2_O_3_. Table 1 lists the specific chemical compositions. In particular, the chemical composition of the raw materials in Table 1 is obtained by XRF testing and is provided by the material supplier.
(b) Metakaolin (MK) is an Aluminum Source Additive in Tests. It is a highly active mineral mainly composed of amorphous aluminum silicate that is formed by calcining superfine kaolin at low temperatures [17]. The MK was provided by Jinshan mineral product processing plant in Henan Province, China. This white powder, through a 4000-mesh sieve, has an activity index greater than 110 and a loss on ignition of 0.29%. The content of the active components SiO_2_ and Al_2_O_3_ in MK is more than 90%, and its main chemical composition is shown in Table 1.
(c) Sodium hydroxide (NaOH), white flake solid, pure in quality grade analysis, provided by Hengyuan chemical factory in Jiangsu, China.
(d) Sodium silicate (Na_2_SiO_3_), industrial grade powdery instant sodium silicate with a modulus of 2, provided by Hengyuan chemical plant in Jiangsu, China.

### 2.2. Mix Design and Sample Preparation

To avoid unnecessary intervention combinations, fewer test combinations are used to assess the weight of influence of multiple factors on the mechanical properties of geopolymers [28,29]. In this paper, the orthogonal experimental design (OED) method is used to design the material mix design and prepare samples. From the ratio of the alkali activator and the content of the aluminum regulator, the modulus of the alkali activator (n(SiO_2_)/n(Na_2_O)), the solid-to-liquid ratio of the activator (m(Na_2_O)/m(H_2_O)), the water–cement ratio (m(H_2_O)/m(BFS + MK)) and the metakaolin replacement rate (m(MK)/m(BFS + MK)) were selected as the four factors of orthogonal design.

The setting range of each factor and the control of increasing the step length are as follows: The modulus of the alkali activator (factor A) ranges from 1.1 to 1.7, and the step length is increased by 0.2. The solid-to-liquid ratio of the activator (factor B) ranges from 0.20 to 0.35, and the increasing step length is 0.05; the water–cement ratio (factor C) ranges from 0.30 to 0.45, and the increasing step length is 0.05; the metakaolin replacement rate (factor D) ranges from 0.00 to 0.45, and the step length is increased by 0.15. The factor levels of the orthogonal test are shown in Table 2.

The sample preparation includes four steps: the mixing of the dry materials, alkali activator preparation, pouring and curing. According to the mixture ratio in Table 3, the BFS and MK are first mixed evenly as dry materials, and then the dry material is mixed with the alkali activator solution and stirred for 3 min. Then, the stirred slurry is poured into a standard abrasive tool of 20 mm × 20 mm × 20 mm, fully vibrated and compacted. The samples were cured at room temperature for 24 h and then continued to be cured to the specified age. The curing temperature was (20 ± 2) °C, and the relative humidity was about 65~70%.

### 2.3. Test and Characterization Methods

**Figure 1:**
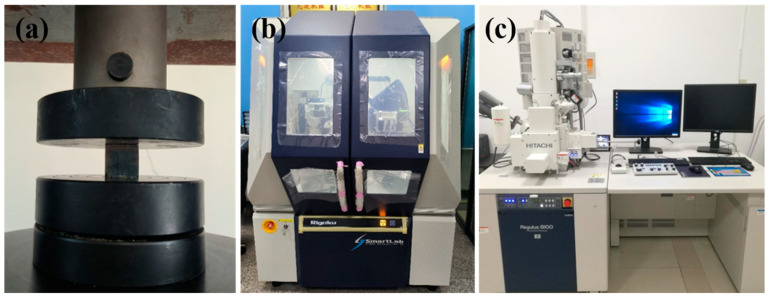
(**a**) Uniaxial compression test; (**b**) XRD test; (**c**) SEM test.

(1) Uniaxial compression test: The uniaxial compressive strength test of the alkali-activated slag-based geopolymer was carried out in the uniaxial compression mode of the SYD0709 (Hangxing Instrument Manufacturing Co., Guangdong, China) Marshall stability tester (Figure 1a). The maximum range of the instrument is 50 KN, the loading rate is controlled to 1 mm/min, and the strength of the sample is tested at 7 d and 28 d. According to the test method standard of the physical and mechanical properties of concrete, the cubic compressive strength R_c_ of the sample is defined as the stress when the sample is destroyed.
(2) XRD test: An Xpert Pro intelligent X-ray diffractometer (PANalytical B.V., Almelo, Netherlands) was used for the phase analysis of alkali-activated slag-based geopolymers (as shown in Figure 1b) with a scanning range of 20°~60°. The glass tube anode type of the machine is Cu, the test speed is set to 5°/min and the step length is 0.02°. The samples used for the XRD test were cured to 28 d, crushed to powder and sieved through 200 mesh.
(3) SEM test: This paper uses the JSM-6501 scanning electron microscope (JEOL, Tokyo, Japan) to observe the samples. The test voltage is 5 kV, and the SE mode is adopted.

## 3. Results and Discussion

### 3.1. Uniaxial Compressive Strength of the Alkali-Activated Slag Sample

In this paper, 16 groups of samples were prepared by the orthogonal experimental design (OED) method, which can quantify the order of contribution of four influencing factors to compressive strength. In each group of experiments shown in Table 3, we prepared eight samples. The uniaxial compression test results of 16 groups of alkali-activated slag samples are shown in Table 4. In particular, the uniaxial compressive strength shown in Table 4 is the average strength of the three samples. The original data of the test can be found in Table S1 in the Supplementary Material.

**Figure 2:**
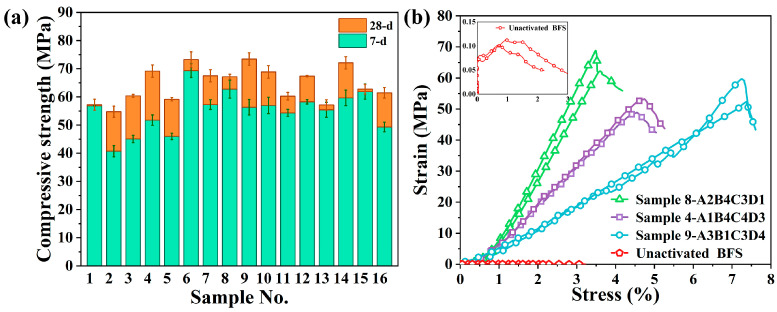
Results of the uniaxial compression test: (**a**) Uniaxial compressive strength of the alkali-activated slag sample cured for 7 and 28 days; (**b**) Uniaxial compression curves of the alkali-activated slag sample.

Figure 2a more intuitively reflects the changing trend of the uniaxial compressive strength of the samples in the form of a histogram. It can be seen that all the samples in the experimental group obtained a considerable compressive strength in the early stage of curing, and the uniaxial compressive strength at 7 days was greater than 40 MPa. This is mainly attributed to the existence of a large number of calcium source precursors in the reaction system, which effectively accelerates the hydration rate of the geopolymer system. Based on the ratio of group 1, we prepared the slag-water samples without an alkali activator. The results of the uniaxial compression test showed that the strength was only 0.1 MPa. This is enough to see the significant effect of alkali-activated modification on the physical and mechanical properties of the sample. Figure 2b is the stress–strain curve of two samples in each group. The original results of the test can be found in Figure S1 in the Supplementary Material. It also shows that the addition of metakaolin significantly increases the ultimate strain of slag specimens. When cured to 28 days, the uniaxial compressive strength of each group is greater than 55 MPa. The strength growth of the geopolymer after 7 days is related to the combined action of many factors [30,31]. However, according to the existing research, the rapid depletion of Ca^2+^ involved in the formation of C(A)SH gel and the slow formation of NASH gel at room temperature are the fundamental reasons for the slow hydration reaction rate of alkali-activated high-calcium system materials in the later stage [32].

### 3.2. Range Analysis of the Uniaxial Compressive Strength

Due to the comprehensive comparability of the orthogonal test, in the range analysis, $T_{Aj}$ represents the sum of the data of all levels under the factor A, and the data changes of ${\bar{T}}_{Aj}$ (the average of the *j*-level data of the factor A) can be generally regarded as being caused by the different levels of the factor A. The range $R_{A}$ (the difference between the maximum and minimum values of ${\bar{T}}_{Aj}$) can be approximately regarded as the degree of change in the whole test caused by the level change of factor A. Thus, the primary and secondary factors of the test can be compared intuitively, and the optimal level of collocation can be found through fewer test groups [33]. The range analysis of the compressive strength of the alkali-activated slag at 7 days and 28 days is shown in Table 5. From the perspective of the mix ratio, the order of sensitivity of the compressive strength to various factors is as follows: the compressive strength at 7 days, the modulus of the alkali activator > the Metakaolin replacement rate > the water–cement ratio > the solid-to-liquid ratio of the activator; the compressive strength at 28 days, the modulus of the alkali activator > the water–cement ratio > the solid-to-liquid ratio of the activator > the Metakaolin replacement rate.

**Figure 3:**
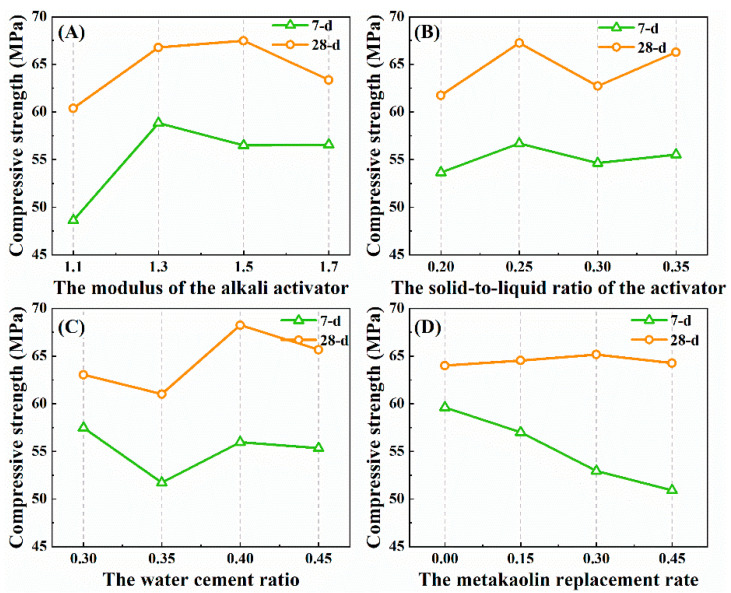
Influence trend of each factor on the strength of the alkali-activated slag sample: (**A**) The modulus of the alkali activator; (**B**) The solid-to-liquid ratio of the activator; (**C**) The water cement ratio; (**D**) The metakaolin replacement rate.

On the basis of Table 5, Figure 3 can more intuitively reflect the influence trend of various factors on the strength of the alkali-activated slag. Among them, for the 7-day age sample, the optimal scheme to obtain a higher compressive strength is A2B2C1D1; for the 28-day age specimens, the optimal scheme is A3B2C3D3. The influence trend of each factor on the strength of the sample is analyzed, and the internal mechanism is as follows:

(a) The modulus of the alkali activator

The alkali activator mainly provides Si-containing components and an alkaline catalytic environment in the slag hydration reaction, and its chemical properties are determined by the modulus. In the system of high-calcium raw materials, the modulus of the alkali activator is too low, and a large amount of OH^−^ will inhibit the dissolution and reaction of CaO, accelerate the formation of Ca(OH)_2_ and slow down the formation rate of C(A)SH gel, resulting in a decrease in the strength of the reaction product [17,34]. When the modulus is too high, the weaker the alkalinity and the greater the viscosity, which is not conducive to the geological polymerization reaction, which also leads to the lower strength of the sample. Therefore, the appropriate modulus is the most critical factor in determining the mechanical properties of alkali-activated high-calcium materials [31].

(b) The solid-to-liquid ratio of the activator

The solid–liquid ratio of the activator reflects the concentration of the effective component Na_2_O·nSiO_2_ in the solution. Figure 3 shows that a higher solid-to-liquid ratio of the activator does not necessarily guarantee a higher strength of the sample. In the alkali-activated reaction, because the silicon-oxygen bond has a larger bond energy than the aluminum–oxygen bond, the rate of ionization of Si ions from the precursor raw materials is slower. At this time, the addition of the silicate alkali activator can provide a large number of Si components for the reaction, and the essence of promoting the geological polymerization process is to ensure the relative coordination of the leaching rate of silicon and aluminum.

(c) The water–cement ratio

As a medium in the dissolution–precipitation process of the alkali-activated reaction [35], the presence of water is conducive to the transport of ions in the reaction system. At the same time, the existing studies believe that the geological polymerization reaction mainly includes three steps. First, the active Si/Al oxide is dissolved in the activator solution; then, the monomer is reconstructed to form an oligomeric structural unit; finally, the structural units are further condensed to form the polymer. In the monomer reconstruction stage with water as a reactant, an appropriate amount of water helps to accelerate the formation of structural units. However, in the polycondensation reaction stage, as one of the reaction products, the water in the system will inhibit the polymerization process [36]. Nevertheless, in the middle and later period of curing, this effect is weaker than that of water on ions transport, so the strength of the sample with a larger water–cement ratio is relatively higher at 28 days.

(d) The Metakaolin replacement rate

Metakaolin is added to the reaction system as an aluminum source admixture. It can be found that the metakaolin replacement rate has a greater effect on the early strength of the alkali-activated slag, while it has little effect on the middle- and later-period strength. Obviously, this is only a surface conclusion. The fundamental reason lies in the different reaction mechanisms. As a low-calcium and high-aluminum raw material, metakaolin mainly participates in the geological polymerization reaction and generates NASH gel under the action of the alkali activator, which is relatively slow. As a high-calcium and low-aluminum material, the hydration reaction product C(A)SH gel of slag is formed more quickly [16]. At the early stage of curing, the product of slag hydration is the main source of sample strength. With the increase in the curing age, the hydration reaction of the slag gradually stops, while the formation of the NASH gel is still slow, and the increase in strength mainly depends on the geological polymerization of the aluminum source materials.

### 3.3. Variance Analysis of Uniaxial Compressive Strength

Range analysis can realize the primary and secondary sorting of each influencing factor so as to clarify the main factors affecting the strength of alkali-activated slag samples. However, in the orthogonal test, the change in the strength index may be attributed to the change in the factor level or the random error of the test, but we cannot distinguish the influence degree of the two. Therefore, it is difficult to accurately explain whether the influence of various factors on the strength of the sample is significant and how significant it is only through range analysis. Therefore, we need to further introduce variance analysis to determine the significance of each factor’s influence on the strength index by calculating the difference between the fluctuation of the strength index caused by each factor and the fluctuation caused by the error.

The compressive strength results of alkali-activated slag samples at 7 days and 28 days were analyzed by variance analysis. The statistical indexes are shown in Table 6. The larger the F value in the table, the higher the influence of the corresponding factors on the compressive strength of the sample. It can be seen that the significant degree of influence of each factor on the early strength of the sample is as follows: At the early stage of curing, the modulus of the alkali activator > the Metakaolin replacement rate > the water–cement ratio > the solid-to-liquid ratio of the activator. In the middle and later periods of curing, the modulus of the alkali activator > the water–cement ratio > the solid-to-liquid ratio of the activator > the Metakaolin replacement rate.

### 3.4. XRD Phase Analysis

The uniaxial compression test results show that the Metakaolin replacement rate plays a key role in the later strength growth of the sample. In order to further analyze the factors affecting the strength of the alkali-activated slag samples, clarify the slag alkali-activated process and reaction products. XRD tests were carried out on some alkali-activated slag samples cured to 28 days, and the main phase composition of the samples was obtained. Among them, sample 1 corresponds to the first group of the orthogonal test, and the raw materials only contain slag. Sample 9 corresponds to the ninth group of the orthogonal test, which has the highest 28-day strength and the highest replacement rate of metakaolin. Sample 12 corresponds to the 12th group of the orthogonal test, and its strength and metakaolin replacement rate are between sample 1 and sample 9.

**Figure 4:**
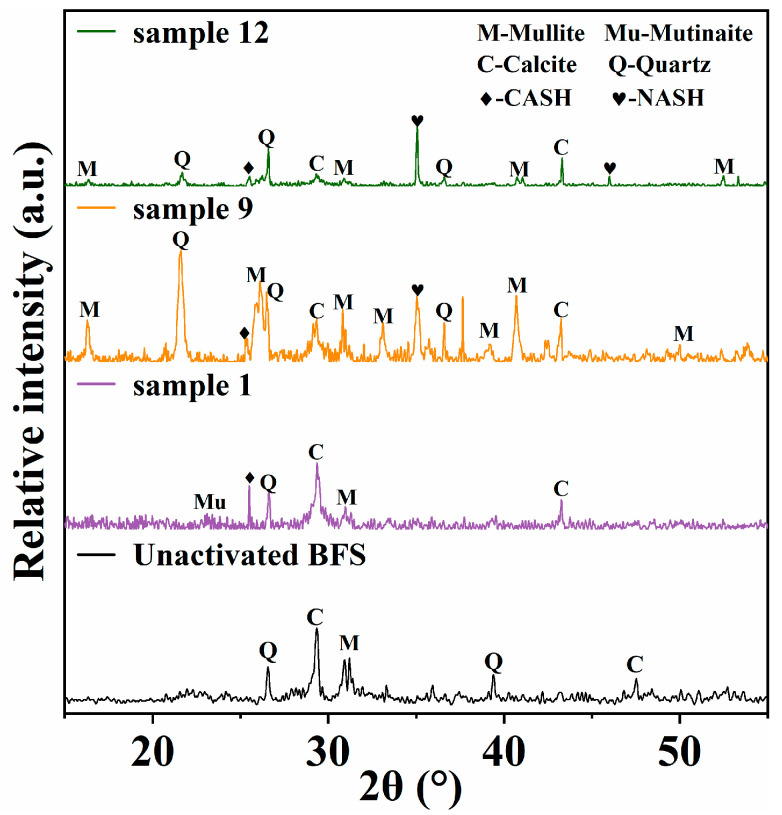
XRD patterns of alkali-activated slag samples.

The XRD patterns of the alkali-activated slag sample are shown in Figure 4. In the inactivated BFS samples, the main phase composition is calcite and quartz. In particular, we can find that, in sample 1, the main phases are calcite and quartz, which mainly come from incompletely reacted slag particles. The diffraction peak near 25.3° can be considered as the main product CASH gel formed by the alkali-activated slag reaction. At the same time, amorphous aluminosilicate products such as mullite and mutinaite were also formed in the reaction system. Compared with sample 1, the XRD test results of sample 9 show that it contains more intermediate mullite. At the same time, the diffraction peak near 35.0° indicates the formation of new NASH gels. This diffraction peak is also captured in sample 12, but the phase of mullite decreased obviously.

The XRD test results further verify the correlation analysis of sample strength. For the alkali-activated reaction of pure slag, the formation of CASH gel contributed to the main strength of the sample. However, this process has been fully carried out in the early stage of curing, so the late strength growth of sample 1 is very small. In contrast, a certain amount of metakaolin is used to replace the slag in order to increase the proportion of aluminum in the reaction system. At this time, a large amount of NASH gel will be formed in the system, which is a good supplement to the late strength growth of the sample [35].

### 3.5. SEM Analysis

**Figure 5:**
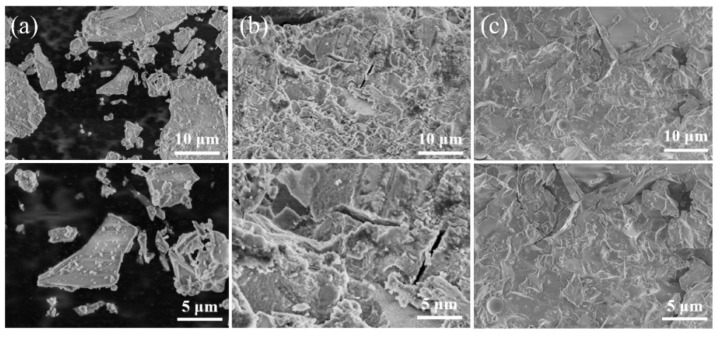
SEM test results: (**a**) The raw material (BFS) for testing; (**b**) sample 1—A1B1C1D1 (pure slag geopolymer sample); (**c**) sample 4—A1B4C4D3 (0.7 slag + 0.3 metakaolin geopolymer sample).

For the samples studied in this paper, the SEM test helps in analyzing the microstructure, the distribution of defects (micro-cracks and micro-holes) and the mineral components’ morphology. Figure 5 shows the results of the SEM test. Figure 5a shows the microstructure of the blast furnace slag powder used in this test. Its micromorphology is flakey or granular with a small particle size. Figure 5b,c show the microstructure of sample 1 and sample 4, respectively. Their ratios are detailed in Table 3. In particular, we can find that the addition of metakaolin limits the development of micro-cracks in the alkali-activated slag material, and the structure of the sample is more compact. Therefore, in the macroscopic properties, the addition of metakaolin can improve the brittleness of alkali-activated slag material significantly.

## 4. Conclusions

In summary, this paper comprehensively studies the effects of the modulus of the alkali activator, the solid-to-liquid ratio of the activator, the water–cement ratio and the Metakaolin replacement rate on the uniaxial compressive strength of the slag-base geopolymer by an orthogonal analysis and XRD test. The primary and secondary degree of the influence of the above factors on the strength of each age of the sample were clarified, and the influence mechanism of each factor was analyzed in depth. The following conclusions were obtained:

(1) The significant degree of influence of each factor on the strength of the sample is as follows: At the early stage of curing, the modulus of the alkali activator > the Metakaolin replacement rate > the water–cement ratio > the solid-to-liquid ratio of the activator. In the middle and later period of curing, the modulus of the alkali activator > the water–cement ratio > the solid-to-liquid ratio of the activator > the Metakaolin replacement rate.
(2) From the point of view of the alkali activator, the alkali activator mainly composed of sodium silicate and sodium hydroxide, on the one hand, serves as a catalyst in the alkali-activated reaction, and, on the other hand, it provides a large number of Si-components in the reaction system. The effect of its mix ratio on the strength of the sample is more complicated. Different levels of the alkali activator modulus, alkali activator solid–liquid ratio and water-cement ratio have little effect on the strength growth of the sample, so the specific ratio of the alkali activator determines the final strength level of the sample to a certain extent. In addition, through orthogonal analysis, we found that the samples with a higher strength can be prepared by setting the modulus of the alkali activator between 1.3 and 1.5 and the water–cement ratio to about 0.4. The test results also show that a higher content of the alkali activator in the system is not necessarily better. From the perspective of economy, the solid-to-liquid ratio is selected at about 0.25, which can obtain a higher strength at the same time.
(3) From the perspective of element composition, in the alkali-activated slag system with high calcium and low aluminum levels, increasing the proportion of aluminum in the system by increasing the metakaolin replacement rate can significantly promote the late strength and toughness growth of the sample. In this process, the new gel phase NASH generated by the continuous reaction plays a key role.
(4) From the above conclusions, we can prepare the sample according to the expected material properties. For example, to obtain early strength building materials, we can choose pure slag as a raw material. If it is necessary to ensure that the material strength can still increase effectively with time while ensuring high early strength, we can choose to use an appropriate amount of high-aluminum and low-calcium raw materials in the slag system to achieve the desired purpose. This can provide some reference for engineering applications.

## Figures and Tables

**Table 1:** Main chemical composition of raw materials (wt%).

Raw Material	CaO	SiO_2_	Al_2_O_3_	SO_3_	Fe_2_O_3_	MgO
Blast furnace slag (BFS)	34.00	34.50	17.70	1.64	1.03	6.01
Metakaolin (MK)	0.17	55.06	43.02	/	0.76	0.06

**Table 2:** The level of each factor in the orthogonal test.

Level	A (The Modulus of the Alkali Activator)	B (The Solid-to-Liquid Ratio of the Activator)	C (The Water–Cement Ratio)	D (The Metakaolin Replacement Rate)
1	1.1	0.20	0.30	0.00
2	1.3	0.25	0.35	0.15
3	1.5	0.30	0.40	0.30
4	1.7	0.35	0.45	0.45

**Table 3:** Design of the orthogonal experimental table.

Mix No.	Mix Design				Mass Distribution (g)		Alkali Activator (g)		
	A	B	C	D	BFS	MK	Water	Na_2_SiO_3_	NaOH
1-A1B1C1D1	1.1	0.20	0.30	0%	103.23	0.00	30.97	10	3.60
2-A1B2C2D4	1.1	0.25	0.35	45%	77.86	63.71	49.55	20	7.19
3-A1B3C3D2	1.1	0.30	0.40	15%	87.75	15.48	41.29	20	7.19
4-A1B4C4D3	1.1	0.35	0.45	30%	55.06	23.60	35.39	20	7.19
5-A2B1C2D3	1.3	0.20	0.35	30%	104.82	44.92	52.41	20	4.73
6-A2B2C1D2	1.3	0.25	0.30	15%	118.79	20.96	41.93	20	4.73
7-A2B3C4D4	1.3	0.30	0.45	45%	42.70	34.94	34.94	20	4.73
8-A2B4C3D1	1.3	0.35	0.40	0%	74.87	0.00	29.95	20	4.73
9-A3B1C3D4	1.5	0.20	0.40	45%	62.45	51.10	45.42	20	2.93
10-A3B2C4D1	1.5	0.25	0.45	0%	80.75	0.00	36.34	20	2.93
11-A3B3C1D3	1.5	0.30	0.30	30%	70.66	30.28	30.28	20	2.93
12-A3B4C2D2	1.5	0.35	0.35	15%	63.03	11.12	25.95	20	2.93
13-A4B1C4D2	1.7	0.20	0.45	15%	75.70	13.36	40.08	20	1.55
14-A4B2C3D3	1.7	0.25	0.40	30%	56.11	24.05	32.06	20	1.55
15-A4B3C2D1	1.7	0.30	0.35	0%	76.34	0.00	26.72	20	1.55
16-A4B4C1D4	1.7	0.35	0.30	45%	41.99	34.35	22.90	20	1.55

**Table 4:** The results of the uniaxial compression test.

Mix No.	Mix Design				Compressive Strength (MPa)	
	A	B	C	D	7 Days	28 Days
1-A1B1C1D1	1.1	0.20	0.30	0%	56.88	57.25
2-A1B2C2D4	1.1	0.25	0.35	45%	40.75	54.75
3-A1B3C3D2	1.1	0.30	0.40	15%	45.08	60.38
4-A1B4C4D3	1.1	0.35	0.45	30%	51.75	69.17
5-A2B1C2D3	1.3	0.20	0.35	30%	46.00	59.17
6-A2B2C1D2	1.3	0.25	0.30	15%	69.33	73.25
7-A2B3C4D4	1.3	0.30	0.45	45%	57.25	67.50
8-A2B4C3D1	1.3	0.35	0.40	0%	62.75	67.17
9-A3B1C3D4	1.5	0.20	0.40	45%	56.33	73.42
10-A3B2C4D1	1.5	0.25	0.45	0%	57.00	68.88
11-A3B3C1D3	1.5	0.30	0.30	30%	54.33	60.25
12-A3B4C2D2	1.5	0.35	0.35	15%	58.25	67.38
13-A4B1C4D2	1.7	0.20	0.45	15%	55.42	57.17
14-A4B2C3D3	1.7	0.25	0.40	30%	59.67	72.08
15-A4B3C2D1	1.7	0.30	0.35	0%	61.88	62.75
16-A4B4C1D4	1.7	0.35	0.30	45%	49.33	61.42

**Table 5:** Range analysis table.

Factors	Compressive Strength of 7 Days				Compressive Strength of 28 Days			
	A	B	C	D	A	B	C	D
$T_{i1}$	194.458	214.625	229.875	238.500	241.542	247.000	252.167	256.042
$T_{i2}$	235.333	226.750	206.875	228.083	267.083	268.958	244.042	258.167
$T_{i3}$	225.917	218.542	223.833	211.750	269.917	250.875	273.042	260.667
$T_{i4}$	226.292	222.083	221.417	203.667	253.417	265.125	262.708	257.083
${\bar{T}}_{i1}$	48.615	53.656	57.469	59.625	60.385	61.750	63.042	64.010
${\bar{T}}_{i2}$	58.833	56.688	51.719	57.021	66.771	67.240	61.010	64.542
${\bar{T}}_{i3}$	56.479	54.635	55.958	52.938	67.479	62.719	68.260	65.167
${\bar{T}}_{i4}$	56.573	55.521	55.354	50.917	63.354	66.281	65.677	64.271
$R_{i}$	10.219	3.031	5.750	8.708	7.094	5.490	7.250	0.625

**Table 6:** Variance analysis table on 7 days and 28 days of compressive strength.

Factors	Compressive Strength of 7 Days				Compressive Strength of 28 Days			
	Sum of Squares	Degree of Freedom	Mean Square Sum	F	Sum of Squares	Degree of Freedom	Mean Square Sum	F
A	240.27	3	80.09	0.90	129.10	3	43.03	0.59
B	19.98	3	6.66	0.07	85.65	3	28.55	0.39
C	71.37	3	23.79	0.27	119.32	3	39.77	0.54
D	185.36	3	61.79	0.69	2.95	3	0.98	0.01
Error	267.93	3	89.31	\	219.34	3	73.11	\
T	784.91	15	F(3,3)0.10 = 5.36		556.37	15	F(3,3)0.10 = 5.36	

## Data Availability

The data presented in this study are available on request from the corresponding author.

## Supplementary Materials

The following supporting information can be downloaded at: https://www.mdpi.com/article/10.3390/ma15248795/s1, Figure S1: The stress-strain curve of two samples in some group. Table S1: The original data of uniaxial compression test.
