# Supplementary material for: Orthogonal Experimental Study on the Factors Affecting the Mechanical Properties of Alkali-Activated Slag Materials
Source: Materials (Basel). 2022 Dec 9;15(24):8795. doi: 10.3390/ma15248795 (PMC9782184; doi:10.3390/ma15248795)
Supplement: Supplementary file 1 [file materials-15-08795-s001.zip › materials-2061760-supplementary.pdf]

*materials*

MDPI

# Supplementary Information

## 1. The original data of uniaxial compression test

In each group of experiments shown in Table 3, we prepared 8 samples. Table S1 records the original data of uniaxial compression specimens. Fig. S1 is the stress-strain curve of two samples in some group.

**Table S1.** The original data of uniaxial compression test.

| Mix No. | Compressive Strength of 7 Days | | | | | Compressive Strength of 28 Days | | | | |
|---|---|---|---|---|---|---|---|---|---|---|
| | SM -A | SM-B | SM-C | Average Value | Standard Error | SM-A | SM-B | SM-C | Average Value | Standard Error |
| 1-A1B1C1D1 | 56.85 | 57.00 | 56.75 | 56.875 | 0.07 | 57.00 | 53.25 | 61.50 | 57.250 | 1.95 |
| 2-A1B2C2D4 | 42.25 | 36.00 | 44.00 | 40.750 | 1.98 | 54.50 | 59.00 | 50.75 | 54.750 | 1.99 |
| 3-A1B3C3D2 | 48.25 | 43.25 | 43.75 | 45.083 | 1.30 | 60.18 | 61.55 | 59.40 | 60.375 | 0.51 |
| 4-A1B4C4D3 | 47.75 | 52.00 | 55.50 | 51.750 | 1.83 | 64.00 | 70.50 | 73.00 | 69.167 | 2.19 |
| 5-A2B1C2D3 | 48.75 | 44.00 | 45.25 | 46.000 | 1.16 | 60.25 | 57.75 | 59.50 | 59.167 | 0.60 |
| 6-A2B2C1D2 | 64.25 | 69.25 | 74.50 | 69.333 | 2.42 | 78.00 | 75.00 | 66.75 | 73.250 | 2.75 |
| 7-A2B3C4D4 | 60.25 | 57.25 | 54.25 | 57.250 | 1.73 | 67.50 | 72.25 | 62.75 | 67.500 | 2.17 |
| 8-A2B4C3D1 | 56.30 | 69.75 | 62.75 | 62.750 | 3.18 | 68.50 | 65.00 | 68.00 | 67.167 | 0.89 |
| 9-A3B1C3D4 | 55.75 | 50.25 | 62.00 | 56.333 | 2.77 | 68.50 | 73.75 | 78.00 | 73.417 | 2.24 |
| 10-A3B2C4D1 | 57.75 | 67.50 | 56.25 | 57.000 | 2.88 | 68.58 | 64.3 | 73.75 | 68.875 | 2.24 |
| 11-A3B3C1D3 | 57.25 | 53.50 | 52.25 | 54.333 | 1.23 | 58.50 | 63.50 | 58.75 | 60.250 | 1.34 |
| 12-A3B4C2D2 | 58.75 | 56.75 | 59.75 | 58.250 | 0.78 | 67.38 | 67.25 | 67.50 | 67.375 | 0.07 |
| 13-A4B1C4D2 | 59.75 | 49.00 | 57.50 | 55.417 | 2.67 | 55.00 | 54.50 | 62.00 | 57.167 | 1.86 |
| 14-A4B2C3D3 | 53.00 | 64.25 | 61.75 | 59.667 | 2.78 | 77.00 | 67.75 | 71.50 | 72.083 | 2.19 |
| 15-A4B3C2D1 | 52.00 | 62.50 | 61.25 | 61.875 | 2.70 | 65.75 | 63.75 | 58.75 | 62.750 | 1.73 |
| 16-A4B4C1D4 | 53.50 | 47.25 | 47.25 | 49.333 | 1.70 | 66.00 | 58.00 | 60.25 | 61.417 | 1.86 |

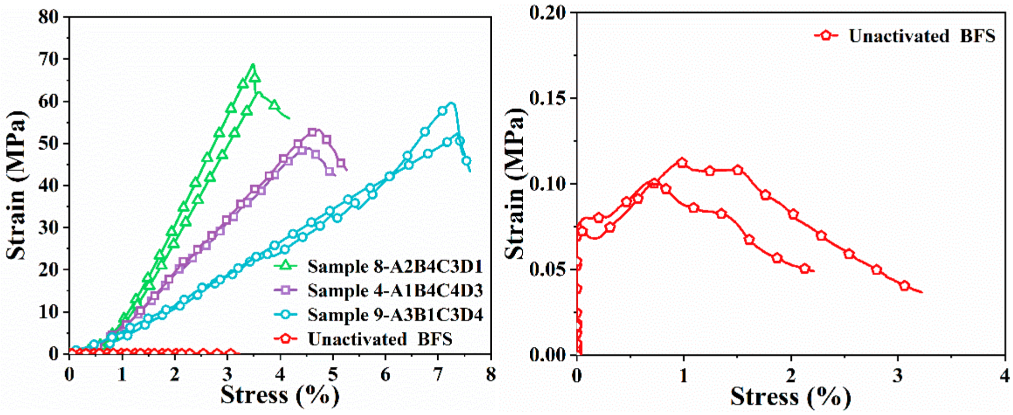


**Figure S1.** The stress-strain curve of two samples in some group.
